# Exposure to e-cigarette advertisements and non-advertising content in relation to use behaviors and perceptions among US and Israeli adults

**DOI:** 10.18332/tpc/173558

**Published:** 2023-11-29

**Authors:** Zongshuan Duan, Lorien C. Abroms, Yuxian Cui, Yan Wang, Cassidy R. LoParco, Hagai Levine, Yael Bar-Zeev, Amal Khayat, Carla J. Berg

**Affiliations:** 1 Department of Population Health Sciences, School of Public Health, Georgia State University, Atlanta, United States; 2 Department of Prevention and Community Health, Milken Institute School of Public Health, George Washington University, Washington, United States; 3 George Washington Cancer Center, George Washington University, Washington, United States; 4 Braun School of Public Health and Community Medicine, Faculty of Medicine, The Hebrew University of Jerusalem and Hadassah Medical Centre, Jerusalem, Israel

**Keywords:** electronic cigarettes, advertising, non-advertising promotion, susceptibility, perceptions

## Abstract

**INTRODUCTION:**

As e-cigarette marketing strategies diversify, it is important to examine exposure to and impact of e-cigarette advertisements and non-advertising content (e.g. on social media) via multiple media channels among adults in different regulatory contexts.

**METHODS:**

Using 2021 cross-sectional data among 2222 adults in the US (n=1128) and Israel (n=1094), multivariable regression examined past-month e-cigarette advertisement and non-advertising content exposure in relation to past-month e-cigarette use (logistic regression), as well as use intentions and risk perceptions (linear regressions), controlling for sociodemographics and tobacco use.

**RESULTS:**

Overall, 20.3% reported past-month e-cigarette use (15.5% US, 25.2% Israel), 46.1% any advertisement exposure (28.7% digital media, 25.2% traditional media, 16.8% retail settings), and 34.1% any non-advertising exposure (19.4% social media, 13.6% websites, 12.3% movie/television/theater, 5.8% radio/podcasts). Exposure to digital media advertisements (AOR=1.95; 95% CI: 1.42–2.66), traditional media advertisements (AOR=2.00; 95% CI=1.49–2.68), and social media non-advertising (AOR=1.72; 95% CI: 1.25–2.36) correlated with e-cigarette use. Exposure to traditional media advertisements (β=0.23; 95% CI: 0.08–0.38) and social media non-advertising (β=0.26; 95% CI: 0.09–0.43) correlated with use intentions. Exposure to digital media advertisements (β= -0.32; 95% CI: -0.57 – -0.08), retail setting advertisements (β= -0.30; 95% CI: -0.58 – -0.03), and radio/podcast non-advertising (β= -0.44; 95% CI: -0.84 – -0.03) correlated with lower perceived addictiveness. Radio/podcast non-advertising exposure (β= -0.50; 95% CI: -0.84 – -0.16) correlated with lower perceived harm. However, retail setting advertisement exposure was associated with e-cigarette non-use (AOR=0.61; 95% CI: 0.42–0.87), and traditional media advertisement (β=0.38; 95% CI: 0.15–0.61) and social media non-advertising exposure (β=0.40; 95% CI: 0.14–0.66) correlated with greater perceived addictiveness.

**CONCLUSIONS:**

E-cigarette-related promotional content exposure across media platforms impacts perceptions and use, thus warranting regulation.

## INTRODUCTION

Over the past decade, the popularity of e-cigarettes has increased worldwide. Past research indicates that e-cigarette marketing exposure may influence e-cigarette perceptions, use patterns, and use intentions among adults^1-3^. Thus, better understanding of how and where e-cigarettes are promoted, is crucial. For example, paid advertising of e-cigarettes has promoted them as fashionable, exploiting their sleek designs and various flavors, which appeal to young people^4^. Moreover, e-cigarettes are marketed as a harm-reduced alternative to combustible cigarettes^5^, and to support cigarette smoking cessation, despite mixed evidence of their effects for cigarette cessation^6,7^. Notably, e-cigarettes contain various chemicals that can potentially harm the respiratory system, primarily through oxidative stress and inflammation^8^. However, these risks are often inadequately disclosed in marketing messages. Thus, these messages likely influence who uses e-cigarettes (e.g. young people) and why (e.g. social appeal, low perceived risk).

Where e-cigarettes are promoted is equally critical to understanding their impact. E-cigarette companies initially used traditional media channels, such as print, television, and radio, to promote their products^9^. However, in recent years, companies have shifted their focus to aggressively promote their products through various forms of advertisements and non-advertising promotion, including product reviews, on digital media channels such as official and affiliated accounts, and paid sponsors or social influencers^10,11^.

Previous studies on e-cigarette marketing have primarily focused on young adults^1-3^ (with limited research on older adults^12^) and exposure to paid advertising^1-3^. This neglects assessing the larger population impact and how various channels of exposure contribute to that impact^13,14^. Exposure to e-cigarette promotion via digital media has been shown to longitudinally predict e-cigarette use behaviors among young adults^1-3^ and older adults^12^, and sponsored marketing via social and digital media channels have also been shown to influence e-cigarette use^15^. For example, e-cigarette-related posts made by sponsored users on social media have been shown to be perceived as more trustworthy and authentic than posts made by brands’ official accounts or traditional advertising^15^, underscoring the need to better understand the impacts of exposure e-cigarette related content from various sources including those outside of traditional paid advertising.

Given the important role e-cigarette promotion, including paid advertising and from other sources, tobacco control efforts have included the adoption of various policies to regulate e-cigarette marketing^16^. For instance, a recent literature review summarizing cross-country variations in e-cigarette marketing regulations indicated that only a few countries (e.g. Gambia, Honduras) prohibit all forms of e-cigarette advertisements, promotion, and sponsorships^16^.

Despite the rapidly evolving regulatory environment and marketing tactics, little is known about exposure to e-cigarette advertisements and non-advertising content on various types of media channels among the adult population, or its association with e-cigarette use behaviors, intentions, and related perceptions. Furthermore, most previous studies occurred in the US, with few documenting the potential differences in exposure to e-cigarette marketing across countries.

Thus, this study explores exposure to e-cigarette advertisements and non-advertising content and its impacts among adults in the US and Israel, where rates of e-cigarette use are high and marketing restrictions have similarities but also distinctions. The prevalence of past-month e-cigarette use was 4.5% among US adults in 2021^17^ and 10.1% among Israeli adults in 2022^18^. In the US, traditional cigarette advertising has been banned on radio and television since 1971^10^. However, e-cigarette advertising was unregulated in the US until the Food and Drug Administration (FDA) implemented the 2016 deeming rule, which expanded FDA’s regulatory purview to cover all tobacco products including e-cigarettes. This required e-cigarettes to be regulated as a tobacco product^19^, thus restricting e-cigarette marketing to reduce youth exposure and misleading information conveyed to consumers (e.g. regarding health risks)^20-22^. Nonetheless, e-cigarette advertising continues to be disseminated via television, print media, retail settings, and online^23^. In Israel, most forms of e-cigarette advertising and promotion are prohibited, with some exceptions including via newspapers, at tobacco and vaping speciality shops, and via print to consenting consumers aged >21 years^16^.

Research advancing our understanding of e-cigarette promotion is needed, in particular given: 1) the shift of e-cigarette promotion via digital advertising and non-advertising promotion methods^11^, which has been shown to influence e-cigarette use^2,3,15,24^; 2) the limited research on the broader population of adults^12^ (beyond young adults, who have been the primary focus of research^1-3^); and 3) the need to better understand e-cigarette promotion and its impact in different sociopolitical contexts, for example, across countries with different tobacco control contexts and e-cigarette markets^25^. Thus, this study examined associations between e-cigarette advertisement and non-advertising content exposures and the outcomes of e-cigarette use, use intentions, endorsement (i.e. perceived positivity of e-cigarette information exposed to), and risk perceptions (i.e. addictiveness, harm) among US and Israeli adults. We hypothesized that e-cigarette advertising and non-advertising exposure via different media channels would be positively associated with e-cigarette use, use intentions, and endorsement, and negatively associated with risk perceptions among US and Israeli adults.

## METHODS

### Study design and sample

Data used in this cross-sectional study were collected by Ipsos through an online questionnaire survey conducted in the US and Israel, October – December 2021. Information on the study design and sampling strategies is detailed elsewhere^26^ but summarized here. Eligible participants were citizens of the respective countries, aged 18–45 years, and able to speak English (US) or Hebrew or Arabic (Israel). Target sample size (100 per country) and composition were based on power analyses to detect small to medium effects with relation to tobacco use outcomes among key sociodemographic groups (i.e. by sex, racial/ethnic group). Purposive sampling was used to achieve about 40% tobacco users and representation by sex and racial/ethnic group (US: 45% White, 25% Black, 15% Asian, and 15% Hispanic; Israel: 80% Jewish and 20% Arab).

In the US, the survey primarily employed KnowledgePanel® (KP), a web panel using probability sampling. Recruitment involved random digit dialing and address-based sampling. KP members received cash incentives (about $5 for a 25-minute survey). Out of 4960 recruited panelists, 2397 (48.3%) passed eligibility screening, and 1095 (45.7%) completed the survey. To reach specific subgroup recruitment goals, Ipsos recruited a convenience sample of US adults reporting Asian race and tobacco use (via banner ads, web pages, and emails). Among 353 screened individuals, 33 (9.3%) were eligible and completed the survey. In Israel, an opt-in sample was used, mirroring the US approach. Out of 2970 individuals who completed eligibility screening and were eligible, 1094 (36.8%) completed the survey. The final sample included 2222 participants (US: n=1128; Israel: n=1094).

This study is reported following the Strengthening the Reporting of Observational Studies in Epidemiology (STROBE) reporting guideline.

### Measures

Measures were adapted from various sources, particularly international surveillance surveys (e.g. International Tobacco Control Policy Evaluation [ITC] Project^27^, Global Adult Tobacco Survey [GATS]^28^), and the full survey took about 20 minutes to complete.

*Independent variables*

The independent variables were e-cigarette advertisement and non-advertising content exposure.

Advertisement exposure

This was assessed by asking, ‘In the last 30 days, have you noticed vaping product (or e-cigarette) advertisements in any of the following places: 1) websites (e.g. pop-up ads); 2) social media (e.g. Facebook, Instagram, or Twitter); 3) inside stores that sell cigarettes and other tobacco products; 4) outside stores that sell cigarettes and other tobacco products (including on signs in windows, visible from the outside); 5) specialty stores that sell vaping products; 6) television; 7) radio; 8) posters, billboards, etc.; 9) newspapers or magazines; 10) direct mail; and 11) email’^27,28^. We recategorized these into 3 dichotomous variables: 1) digital media (i.e. any exposure via websites, social media, direct mail, email); 2) retail (i.e. any exposure inside or outside tobacco shops or vape shops); and 3) traditional media (i.e. any exposure via television, radio, posters/billboards, newspapers/magazines)^27,28^.

Non-advertising content exposure

This was assessed by asking, ‘Outside of advertisements, in the last 30 days, have you noticed vaping products (or e-cigarettes) being referenced, used, or portrayed in any of the following places: 1) movies, television, or theater; 2) radio or news podcasts; 3) websites; and 4) social media’^27,28^.

In addition, we created variables indicating the number of different types of media channels that participants experienced advertisement exposure (range: 0–11) and non-advertising content exposure (range: 0–4) for the purposes of descriptive analysis and sensitivity analysis (i.e. to examine robustness of the findings using the categorical exposure variables).

*Outcome variables*

These were e-cigarette use, use intentions, endorsement, and risk perceptions. Lifetime e-cigarette use was assessed by asking, ‘In your lifetime, have you ever used e-cigarettes, vaping products, or other electronic nicotine delivery devices (excluding IQOS or similar products)?’. Among those reporting lifetime use, current e-cigarette use was assessed by asking, ‘In the past 30 days, how many days have you used e-cigarettes?’ (0–30 recategorized as yes ≥1; no =0)^27,28^. E-cigarette use intentions were assessed by asking, ‘How likely are you to try or continue to use e-cigarettes in the next year?’ (1 = not at all, to 5 = very)^27,28^. E-cigarette endorsement was assessed by asking, ‘Thinking about all you have seen and read about vaping devices, from all sources, would you say the information has been? (1 = mostly negative, to 5 = mostly positive)’. E-cigarette risk perceptions included: 1) perceived addictiveness, ‘How addictive do you think e-cigarettes, vaping products, or other electronic nicotine delivery systems (such as Juul) are? (1 = not at all addictive, to 7 = extremely addictive)’; and 2) perceived harm, ‘How harmful do you think e-cigarettes, vaping products, or other electronic nicotine delivery systems (such as Juul) are? (1 = not at all harmful, to 7 = extremely harmful)’^27,28^.

*Covariates*

Covariates included country, age, sex, sexual orientation, race/ethnicity, education level, relationship status, children in the home, and past-month use of e-cigarettes, cigarettes, and other tobacco products (i.e. heated tobacco products, hookah, cigar, pipe, smokeless tobacco).

### Statistical analysis

Data management and analysis were conducted using Stata 15.1 (StataCorp). First, descriptive analysis characterized participants, and bivariate analyses (i.e. chi-squared tests for categorical variables; t-tests, analysis of variance or Pearson correlation coefficients for continuous variables) assessed preliminary associations between the predictors of interest and covariates and the outcomes (past-month e-cigarette use, use intentions, endorsement, risk perceptions). Second, multivariable binary logistic regression analyses assessed associations between advertisement exposure and past-month use of e-cigarettes, and multivariable linear regression analyses assessed associations between advertisement exposure and use intentions, endorsement, and risk perceptions. Regression models adjusted for key sociodemographics (i.e. country, age, sex, sexual orientation, education level) and tobacco use (e-cigarettes, cigarettes, other tobacco products). Multiplicative interaction terms between country and the exposure variables were tested for all models. Finally, we conducted sensitivity analysis using sum scores of advertisement exposure to examine the robustness of our findings. All statistical tests were 2-tailed with significance level set at α=0.05.

## RESULTS

### Participant characteristics

Shown in Table 1, of the 2222 participants, 24.6% were aged 18–25 years, 37.4% aged 26–35 years, and 38.0% aged >35 years; 49.8% were female; 15.1% identified as sexual orientation minority; and 50.1% had a college degree or higher. Past-month e-cigarette use in this sample was 20.3%, and average use intentions, endorsement, and perceived addictiveness and harm were 1.96 (SD=1.81), 2.82 (SD=1.12), 5.25 (SD=2.03), and 5.65 (SD=1.74), respectively.

**Table 1:** Bivariate analyses of cross-sectional data examining exposure to e-cigarette promotions and covariates in relation to current e-cigarette use, use intentions, endorsement, and risk perceptions among US and Israeli adults in 2021 (N=2222)

	*Overall*	*Current e-cigarette use*			*E-cigarette use intentions*		*E-cigarette endorsement*		*Perceived addictiveness*		*Perceived harm*	
		*No*	*Yes*									
	*n (%) or M (SD)^§^*	*n (%) or M (SD)^§^*	*n (%) or M (SD)^§^*	*p*	*M (SD) or Coef.^§^*	*p*	*M (SD) or Coef.^§^*	*p*	*M (SD) or Coef.^§^*	*p*	*M (SD) or Coef.^§^*	*p*
**Overall**	2222 (100)	1745 (79.7)	445 (20.3)		1.96 (1.81)		2.82 (1.12)		5.25 (2.03)		5.65 (1.74)	
**Past-month e-cigarette ad exposure^a^**												
**Number of media channels** (0–11)^§^	1.11 (1.68)	0.87 (1.50)	2.10 (2.00)	**<0.001**	0.26	**<0.001**	0.09	**<0.001**	-0.04	**0.087**	-0.09	**<0.001**
**Any ad exposure**												
Yes	985 (46.1)	640 (37.8)	345 (78.4)	**<0.001**	2.61 (2.11)	**<0.001**	2.97 (1.16)	**<0.001**	5.02 (2.04)	**<0.001**	5.36 (1.76)	**<0.001**
No	1150 (53.9)	1055 (62.2)	95 (21.6)		1.42 (1.28)		2.69 (1.07)		5.51 (1.94)		5.93 (1.62)	
**Digital media**												
Yes	627 (28.7)	376 (21.6)	251 (56.5)	**<0.001**	2.80 (2.14)	**<0.001**	3.06 (1.12)	**<0.001**	4.89 (2.03)	**<0.001**	5.31 (1.79)	**<0.001**
No	1558 (71.3)	1365 (78.4)	193 (43.5)		1.63 (1.53)		2.73 (1.10)		5.40 (2.01)		5.78 (1.70)	
**Traditional media**												
Yes	530 (25.2)	340 (20.0)	190 (46.5)	**<0.001**	2.67 (2.18)	**<0.001**	2.93 (1.17)	**0.005**	5.28 (1.92)	0.763	5.48 (1.64)	**0.004**
No	1577 (74.9)	1358 (80.0)	219 (53.6)		1.68 (1.55)		2.78 (1.10)		5.24 (2.08)		5.73 (1.77)	
**Retail settings**												
Yes	366 (16.8)	241 (13.8)	125 (28.2)	**<0.001**	2.57 (2.01)	**<0.001**	2.98 (1.15)	**0.003**	4.85 (2.06)	**<0.001**	5.28 (1.84)	**<0.001**
No	1819 (83.3)	1500 (86.2)	319 (71.9)		1.84 (1.74)		2.79 (1.11)		5.34 (2.01)		5.72 (1.71)	
**Past-month e-cigarette non-ad exposure**												
**Number of media channels** (0–4)^§^	0.51 (0.82)	0.37 (0.72)	1.04 (0.97)	**<0.001**	0.32	**<0.001**	0.11	**<0.001**	-0.10	**<0.001**	-0.14	**<0.001**
**Any non-ad exposure**												
Yes	744 (34.9)	452 (26.7)	292 (66.5)	**<0.001**	2.81 (2.14)	**<0.001**	3.02 (1.17)	**<0.001**	4.95 (2.03)	**<0.001**	5.26 (1.80)	**<0.001**
No	1389 (65.1)	1242 (73.3)	147 (33.5)		1.52 (1.41)		2.71 (1.08)		5.46 (1.97)		5.88 (1.62)	
**Movie, television, or theater**												
Yes	267 (12.3)	172 (9.9)	95 (21.4)	**<0.001**	2.70 (2.09)	**<0.001**	2.95 (1.24)	0.042	4.90 (2.10)	**0.003**	5.27 (1.90)	**<0.001**
No	1913 (87.8)	1565 (90.1)	348 (78.6)		1.86 (1.74)		2.80 (1.10)		5.30 (2.02)		5.70 (1.71)	
**Radio, news podcasts**												
Yes	126 (5.8)	75 (4.3)	51 (11.5)	**<0.001**	2.95 (2.05)	**<0.001**	2.93 (1.12)	**0.240**	4.52 (2.07)	**<0.001**	4.80 (1.87)	**<0.001**
No	2054 (94.2)	1662 (95.7)	392 (88.5)		1.90 (1.77)		2.81 (1.12)		5.30 (2.02)		5.70 (1.72)	
**Websites**												
Yes	297 (13.6)	164 (9.4)	133 (30.0)	**<0.001**	2.93 (2.15)	**<0.001**	3.02 (1.14)	**0.001**	4.85 (2.05)	**<0.001**	5.15 (1.87)	**<0.001**
No	1883 (86.4)	1573 (90.6)	310 (70.0)		1.81 (1.69)		2.79 (1.11)		5.31 (2.02)		5.72 (1.71)	
**Social media**												
Yes	422 (19.4)	239 (13.8)	183 (41.3)	**<0.001**	2.99 (2.23)	**<0.001**	3.08 (1.15)	**<0.001**	5.15 (1.94)	0.239	5.45 (1.65)	**0.013**
No	1758 (80.6)	1498 (86.2)	260 (58.7)		1.71 (1.59)		2.76 (1.10)		5.28 (2.05)		5.69 (1.76)	
**Past-month tobacco use status**												
**E-cigarettes**												
Yes	445 (20.3)	0 (0)	445 (100)	**<0.001**	4.25 (2.19)	**<0.001**	3.15 (1.10)	**<0.001**	4.95 (1.92)	**<0.001**	5.02 (1.66)	**<0.001**
No	1745 (79.7)	1745 (100)	0 (0)		1.36 (1.05)		2.74 (1.11)		5.35 (2.04)		5.82 (1.71)	
**Cigarettes**												
Yes	676 (31.1)	376 (21.7)	300 (68.0)	**<0.001**	2.98 (2.13)	**<0.001**	3.01 (1.09)	**<0.001**	5.04 (1.90)	**0.001**	5.40 (1.67)	**<0.001**
No	1498 (68.9)	1357 (78.3)	141 (32.0)		1.49 (1.40)		2.73 (1.13)		5.35 (2.08)		5.76 (1.77)	
**Other tobacco products^b^**												
Yes	523 (24.1)	260 (15.1)	263 (59.5)	**<0.001**	3.03 (2.16)	**<0.001**	3.07 (1.11)	**<0.001**	4.72 (2.04)	**<0.001**	5.06 (1.86)	**<0.001**
No	1647 (75.9)	1468 (85.0)	179 (40.5)		1.61 (1.52)		2.74 (1.11)		5.44 (1.98)		5.85 (1.65)	
**Demographics**												
**Country**												
USA	1099 (50.2)	929 (53.2)	170 (38.2)	**<0.001**	1.76 (1.72)	**<0.001**	2.65 (1.12)	**<0.001**	5.42 (2.06)	**<0.001**	5.72 (1.72)	0.056
Israel	1091 (49.8)	816 (46.8)	275 (61.8)		2.16 (1.87)		2.99 (1.10)		5.10 (1.99)		5.58 (1.75)	
**Age** (years)												
18–25	539 (24.6)	399 (22.9)	140 (31.5)	**<0.001**	2.13 (1.92)	**0.028**	2.83 (1.12)	0.604	4.94 (2.16)	**<0.001**	5.50 (1.84)	0.052
26–35	818 (37.4)	655 (37.5)	163 (36.6)		1.96 (1.77)		2.84 (1.11)		5.40 (1.93)		5.66 (1.70)	
36–45	833 (38.0)	691 (39.6)	142 (31.9)		1.86 (1.76)		2.79 (1.12)		5.32 (2.01)		5.73 (1.70)	
**Gender**												
Female	1100 (50.2)	901 (51.6)	199 (44.7)	**0.009**	1.84 (1.74)	**0.001**	2.79 (1.07)	0.228	5.39 (2.04)	**0.001**	5.88 (1.64)	**<.001**
Male	1090 (49.8)	844 (48.4)	246 (55.3)		2.09 (1.87)		2.85 (1.16)		5.11 (2.01)		5.41 (1.81)	
**Sexual orientation**												
Heterosexual	1858 (84.9)	1498 (85.9)	360 (80.9)	0.009	1.92 (1.77)	**0.018**	2.80 (1.11)	0.134	5.29 (1.99)	0.069	5.70 (1.69)	**<0.001**
Minority	331 (15.1)	246 (14.1)	85 (19.1)		2.18 (1.99)		2.91 (1.18)		5.07 (2.21)		5.33 (1.95)	
**Education level**												
< College degree	1092 (49.9)	862 (49.4)	230 (51.7)	0.389	2.04 (1.90)	0.068	2.84 (1.09)	0.345	5.02 (2.18)	**<0.001**	5.54 (1.83)	**0.006**
≥ College degree	1098 (50.1)	883 (50.6)	215 (48.3)		1.89 (1.71)		2.80 (1.15)		5.48 (1.84)		5.75 (1.64)	
**Relationship status**												
Married/cohabitating	1173 (53.6)	924 (53.0)	249 (56.0)	0.257	2.02 (1.85)	0.150	2.81 (1.12)	0.704	5.40 (1.94)	**<0.001**	5.71 (1.68)	**0.062**
Other	1017 (46.4)	821 (47.1)	196 (44.0)		1.90 (1.76)		2.83 (1.12)		5.09 (2.11)		5.57 (1.80)	
**Children aged >18 years in the home**												
Yes	1108 (50.6)	869 (49.8)	239 (53.7)	0.141	2.03 (1.89)	0.080	2.86 (1.10)	0.135	5.28 (2.02)	0.513	5.69 (1.73)	0.214
No	1082 (49.4)	876 (50.2)	206 (46.3)		1.89 (1.71)		2.78 (1.14)		5.22 (2.05)		5.60 (1.75)	

§ For continuous variables, M (SD) reported for first 3 columns and Coeff for other columns.

a Digital media: websites, social media, direct mail, email. Retail: inside or outside tobacco shop, vape shop. Traditional media: television, radio, newspapers/magazines, and posters/billboards.

b Other tobacco includes heated tobacco products, hookah, cigar, pipe, and smokeless tobacco. Boldface indicates p<0.05.

Overall, past-month e-cigarette advertisement exposure via any channel was reported by 46.1%, with 28.7% reporting exposure via digital media, 25.2% traditional media, and 16.8% retail settings. The average number of media channels on which participants saw e-cigarette advertisements in the past 30 days was 1.11 (SD=1.68; range 0–11). Past-month e-cigarette non-advertising content via any channel was reported by 34.9%, with 19.4% reporting exposure via social media, 13.6% websites, 12.3% movie, television, or theater, and 5.8% radio or podcasts. The average number of media channels participants were exposed to e-cigarette non-advertising content in the past 30 days was 0.51 (SD=0.82; range 0–4).

Participant characteristics by country are presented in Supplemental file Tables 1 a and b. Among US participants, 15.5% reported past-month e-cigarette use, and average scores were: 1.76 (SD=1.72) for use intentions, 2.65 (SD=1.12) endorsement, 5.42 (SD=2.05) perceived addictiveness, and 5.71 (SD=1.72) perceived harm. Among Israeli participants, 25.2% reported past-month e-cigarette use, and average scores were: 2.16 (SD=1.87) for use intentions, 2.99 (SD=1.10) endorsement, 5.10 (SD=1.99) perceived addictiveness, and 5.58 (SD=1.75) perceived harm.

### Bivariate and multivariate analysis outcomes

In bivariate analysis, correlates of current e-cigarette use, greater use intentions, more positive endorsement, and lower risk perceptions included being exposed to e-cigarette advertisements and non-advertising content via more channels, any ad or non-ad exposure, and each of the categories of ad exposure and non-ads, with few exceptions (Table 1). E-cigarette, cigarette, and other tobacco users were more likely to report current e-cigarette use and reported greater use intentions, more positive endorsement, and lower risk perceptions.

In multivariable regression (Table 2), advertisement exposure via digital media (AOR=1.95; 95% CI: 1.42–2.66) and traditional media (AOR=2.00; 95% CI: 1.49–2.68) and non-advertising content exposure via social media (AOR=1.72; 95% CI: 1.25–2.36) were significantly associated with higher odds of past-month e-cigarette use, while advertisement exposure via retail setting (AOR=0.61; 95% CI: 0.42–0.87) was associated with lower odds of e-cigarette use. Advertisement exposure via traditional media (β=0.23; 95% CI: 0.08–0.38) and non-advertising content exposure via social media (β=0.26; 95% CI: 0.09–0.43) were associated with greater use intentions. Advertisement exposure via digital media (β=0.15; 95% CI: 0.02–0.28) was associated with more positive endorsement. Regarding perceptions, advertisement exposure via digital media (β= -0.32; 95% CI: -0.57 – -0.08) and retail settings (β= -0.30; 95% CI: -0.58 – -0.03) and non-advertising content exposure via radio/podcast (β= -0.44; 95% CI: -0.84 – -0.03) were associated with lower perceived addictiveness, while advertisement exposure via traditional media (β=0.38; 95% CI: 0.15–0.61) and non-advertising content exposure via social media (β=0.40; 95% CI: 0.14–0.66) were associated with greater perceived addictiveness. Non-advertising content exposure via radio/podcast (β= -0.50; 95% CI: -0.84 – -0.16) was associated with lower perceived harm, while exposure via social media (β=0.24; 95% CI: 0.02–0.47) was associated with greater perceived harm. Multivariable regression results by country are presented in Supplemental file Tables 2 a and b.

**Table 2:** Multivariable regression analyses of cross-sectional data examining exposure to e-cigarette promotions and covariates in relation to current e-cigarette use, use intentions, endorsement, and risk perceptions among US and Israeli adults in 2021 (N=2222)

	*Current e-cigarette use*		*E-cigarette use intentions*		*E-cigarette endorsement*		*Perceived addictiveness*		*Perceived harm*	
	*AOR*	*95% CI*	*β*	*95% CI*	*β*	*95% CI*	*β*	*95% CI*	*β*	*95% CI*
**Past-month e-cigarette ad exposure**										
Digital media (Ref: No)	**1.95**	**1.42–2.66**	0.15	-0.02–0.31	**0.15**	**0.02–0.28**	**-0.32**	**-0.57 – -0.08**	-0.16	-0.37–0.04
Traditional media (Ref: No)	**2.00**	**1.49–2.68**	**0.23**	**0.08–0.38**	0.03	-0.09–0.16	**0.38**	**0.15–0.61**	0.04	-0.15–0.24
Retail settings (Ref: No)	**0.61**	**0.42–0.87**	-0.08	-0.26–0.10	0.05	-0.10–0.20	**-0.30**	**-0.58 – -0.03**	-0.05	-0.28–0.18
**Past-month e-cigarette non-ad exposure**										
Movie, television, or theater (Ref: No)	1.02	0.70–1.48	0.14	-0.05–0.33	-0.02	-0.18–0.14	-0.13	-0.42–0.16	-0.11	-0.36–0.13
Radio, news podcasts (Ref: No)	1.00	0.61–1.63	0.11	-0.16–0.38	-0.16	-0.39–0.06	**-0.44**	**-0.84 – -0.03**	**-0.50**	**-0.84– -0.16**
Websites (Ref: No)	1.19	0.83–1.69	0.06	-0.14–0.25	-0.03	-0.19–0.13	-0.13	-0.42–0.17	-0.18	-0.43–0.06
Social media (Ref: No)	**1.72**	**1.25–2.36**	**0.26**	**0.09–0.43**	0.07	-0.07–0.22	**0.40**	**0.14–0.66**	**0.24**	**0.02–0.47**
**Current tobacco use status**										
E-cigarettes (Ref: No)	--	--	**2.42**	**2.25–2.59**	**0.25**	**0.11–0.39**	-0.14	-0.39–0.12	**-0.55**	**-0.77– -0.33**
Cigarettes (Ref: No)	**3.98**	**3.01–5.25**	**0.42**	**0.27–0.57**	0.03	-0.09–0.15	0.02	-0.20–0.24	0.08	-0.10–0.27
Other tobacco products^a^ (Ref: No)	**4.15**	**3.11–5.53**	0.06	-0.11–0.22	0.13	-0.01–0.26	**-0.53**	**-0.77 – -0.28**	**-0.40**	**-0.61– -0.19**
**Demographics**										
USA (Ref: Israel)	1.09	0.81–1.46	-0.08	-0.21–0.05	**-0.33**	**-0.44 – -0.23**	0.18	-0.01–0.37	0.00	-0.16–0.17
**Age** (years) (Ref: 36–45)										
18–25	1.43	1.00–2.04	-0.05	-0.21–0.11	-0.13	-0.26–0.00	-0.15	-0.39–0.09	-0.01	-0.21–0.20
26–35	1.26	0.92–0.72	0.02	-0.12–0.15	0.01	-0.10–0.12	0.14	-0.06–0.34	-0.01	-0.18–0.16
Female (Ref: male)	1.06	0.82–1.39	-0.10	-0.22–0.02	-0.03	-0.13–0.07	0.15	-0.02–0.33	**0.37**	**0.22–0.52**
Sexual orientation minority (Ref: heterosexual)	1.36	0.96–1.93	0.06	-0.11–0.22	0.05	-0.08–0.19	-0.06	-0.31–0.18	**-0.26**	**-0.47– -0.05**
Education level < College (Ref: ≥ College)	1.05	0.79–1.38	0.08	-0.04–0.20	**0.13**	**0.03–0.23**	**-0.49**	**-0.67 – -0.31**	**-0.23**	**-0.39– -0.08**

a Other tobacco includes heated tobacco products, hookah, cigar, pipe, and smokeless tobacco. Boldface indicates p<0.05. In US-specific models, being Black, Asian, or Hispanic was negatively correlated with addictiveness and harm; being Asian was positively correlated with endorsement. In Israel-specific models, being Arabic (vs Jewish) was positively correlated with current e-cigarette use and use intentions.

In terms of tobacco use and sociodemographic correlates, current cigarette and other tobacco use was associated with current e-cigarette use; current e-cigarette and cigarette use was associated with greater use intentions; e-cigarette use, residing in Israel, and having < College education were related to more positive endorsement; other tobacco use and having < College education was associated with lower perceived addictiveness; and e-cigarette and other tobacco use, being male, identifying as sexual minority, and having < College education were associated with lower perceived harm. No significant interactions were found between exposure and country (US vs Israel) in any of the above models, indicating the relations held consistent across the countries.

Sensitivity analyses (Table 3) examining outcomes in relation to the number of media channels participants were exposure to e-cigarette advertisements and non-advertising content indicated that a greater number of channels of advertisement exposure was associated with greater odds of past-month e-cigarette use (AOR=1.19; 95% CI: 1.10–1.29) and a greater number of channels of non-advertising content exposure was associated with greater odds of past-month e-cigarette use (AOR=1.30; 95% CI: 1.10–1.54), greater use intentions (β=0.22; 95% CI: 0.13–0.31), and lower perceived addictiveness (β= -0.15; 95% CI: -0.28 – -0.01) and harm (β= -0.13; 95% CI: -0.24 – -0.01). There were no significant interactions between the exposure and country. Multivariable regression results by country, with the outcome of number of media channels where exposed to e-cigarette content, are presented in Supplemental file Tables 3 a and b.

**Table 3:** Sensitivity analyses: multivariable regression analyses of cross-sectional data examining exposure to e-cigarette promotions per number of channels and covariates in relation to current e-cigarette use, use intentions, endorsement, and risk perceptions among US and Israeli adults in 2021 (N=2222)

	*Current e-cigarette use*		*E-cigarette use intentions*		*E-cigarette endorsement*		*Perceived addictiveness*		*Perceived harm*	
	*AOR*	*95% CI*	*β*	*95% CI*	*β*	*95% CI*	*β*	*95% CI*	*β*	*95% CI*
**Number of media channels**										
Ad exposure (0–10)	**1.19**	**1.10–1.29**	0.01	-0.03–0.05	0.02	-0.01–0.06	0.06	-0.01–0.12	0.02	-0.03–0.08
Non-ad exposure (0–4)	**1.30**	**1.10–1.54**	**0.22**	**0.13–0.31**	0.02	-0.05–0.09	**-0.15**	**-0.28 – -0.01**	**-0.13**	**-0.24 – -0.01**
**Current tobacco use status**										
E-cigarettes (Ref: No)	--	--	**2.47**	**2.30–2.64**	**0.24**	**0.10–0.38**	-0.07	-0.32–0.17	**-0.54**	**-0.74 – -0.33**
Cigarettes (Ref: No)	**4.11**	**3.15–5.36**	**0.45**	**0.31–0.60**	0.06	-0.06–0.18	0.02	-0.20–0.23	0.06	-0.13–0.24
Other tobacco productsa (Ref: No)	**3.89**	**2.96–5.12**	0.05	-0.11–0.21	0.11	-0.02–0.24	**-0.58**	**-0.82 – -0.34**	**-0.45**	**-0.65 – -0.25**
**Demographics**										
USA (Ref: Israel)	1.03	0.78–1.36	-0.10	-0.22–0.03	**-0.34**	**-0.44 – -0.24**	**0.23**	**0.05–0.42**	0.02	-0.14–0.17
**Age** (years) (Ref: 36–45)										
18–25	**1.50**	**1.07–2.11**	-0.06	-0.22–0.10	**-0.13**	**-0.26 – -0.00**	-0.07	-0.31–0.16	-0.02	-0.22–0.18
26–35	1.22	0.91–1.65	0.02	-0.11–0.16	0.01	-0.10–0.12	0.15	-0.05–0.34	-0.02	-0.19–0.15
Female (Ref: male)	1.10	0.85–1.42	-0.10	-0.21–0.02	-0.02	-0.12–0.07	0.17	-0.00–0.34	**0.37**	**0.23–0.52**
Sexual orientation minority (Ref: heterosexual)	1.26	0.90–1.77	0.07	-0.09–0.23	0.04	-0.10–0.17	-0.09	-0.33–0.15	**-0.26**	**-0.46 – -0.06**
Education level < College (Ref: ≥ College)	0.99	0.76–1.29	0.09	-0.03–0.21	**0.11**	**0.01–0.21**	**-0.50**	**-0.68 – -0.32**	**-0.21**	**-0.36 – -0.06**

a Other tobacco includes heated tobacco products, hookah, cigar, pipe, and smokeless tobacco. Boldface indicates p<0.05. In US-specific models, being Black was negatively correlated with current cigarette use, use intentions, addictiveness, and harm; being Asian was positively correlated with endorsement and negatively correlated with addictiveness, and harm; being Hispanic was negatively correlated with addictiveness. In Israel-specific models, being Arabic (vs Jewish) was positively correlated with current e-cigarette use and use intentions.

## DISCUSSION

This cross-sectional study advances research regarding exposure to e-cigarette advertisements and non-advertising content across multiple media channels and their associations with e-cigarette use, use intentions, and related perceptions, and contributes to the limited studies examining such phenomena among adults and/or in cross-country samples. In this sample of adults in the US and Israel, we found that large proportions of participants were exposed to e-cigarette advertisements or non-advertising content, and generally, exposure was associated with greater likelihood of e-cigarette use, greater use intentions, more positive endorsement (i.e. appraisal of information exposed to), and lower perceived risks – albeit with some exceptions (i.e. null or counterintuitive associations). Additionally, findings were consistent across the two countries.

Current results showed that about one-fourth of adults reported past-month exposure to e-cigarette advertising on digital media and traditional media, with about 17% also reporting exposure to retail setting advertisements, suggesting high level of marketing investment and limited restrictions on paid marketing activities in the US and Israel^16,20-22^. Despite laws in Israel prohibiting most forms of advertising, promotion and sponsorship of e-cigarettes^29^ and recent efforts in the US to restrict e-cigarette marketing^30^, these laws may be undermined by inefficient enforcement and/or the industry’s use of marketing strategies to circumvent regulations, particularly on digital media^31^. Perhaps relatedly, exposure to e-cigarette non-advertising content was also concerning, with the highest proportion occurring on social media, likely reflecting the industry’s shift to marketing via social media via companies’ official accounts, affiliated accounts, and/or paid sponsors/influencers, as well as potentially organic user generated content^10^.

The hypothesized associations between e-cigarette advertisement and non-advertising content exposure and e-cigarette use outcomes were generally found, particularly for digital media advertisements (in relation to current use, positive endorsement, lower perceived addictiveness), traditional media advertisements (current use, use intentions), and non-advertising social media content (current use, use intentions). In addition, current findings indicated that exposure to retail setting advertisements and non-advertising content on radio/podcasts was associated with lower risk perceptions. Given the volume of e-cigarette related content on digital media, including paid advertising and unpaid promotion on various platforms including social media^10,11,13,14^, greater efforts are needed to assess the content and context of such content. For example, e-cigarette content on social media often promotes use in a favorable light and lack age and health advisories^32^ and may have immediate effects on use behavior^24^. Because of these impacts, the US FDA has implemented measures to minimize e-cigarette related social media content, including requiring the disclosure of industry use of social media influencers^30^; however, this content remains on major social media channels^13,14^.

Notably, there were several null findings, and some associations were in the unanticipated direction. Advertisement exposure via retail settings was associated with lower odds of current e-cigarette use. Additionally, advertisement exposure via traditional media was associated with greater perceived addictiveness, and non-advertising promotion exposure via social media was associated with greater perceived addictiveness and harm. These counterintuitive findings may be related to the cross-sectional nature of the data and the inclusion of media exposure variables and tobacco use variables in the models, given that bivariate associations were in the anticipated direction. However, other potential reasons might exist. For example, while the counterintuitive finding regarding traditional media is difficult to interpret, especially given that advertisement exposure via traditional media was associated with current e-cigarette use and greater use intentions, it may be that traditional media more likely adhere to required warnings which often include addiction-related content in Israel and must address addictiveness in the US (i.e. ‘WARNING: This product contains nicotine. Nicotine is an addictive chemical’)^33^. The findings regarding social media may be related to a wide variety of ways that participants ‘noticed vaping products (or e-cigarettes) being referenced, used, or portrayed’ in social media (per the assessment), which may include ways that show risky use and/or use among young people, or discuss use-related risks^32^.

Regarding tobacco use and sociodemographic correlates, findings from this study largely align with prior research indicating the common overlap in tobacco use behaviors and use intentions, as well as more favorable perceptions of e-cigarette and tobacco product use among current e-cigarette and tobacco product users^34^. Lower education level was associated with more positive endorsements and lower perceived addictiveness and harm, and being male and identifying as sexual minority were associated with lower perceived harm, findings that reflect previous studies characterizing demographics of adults reporting e-cigarette use^35^.

### Implications

These findings have implications for research and regulatory efforts. Given the e-cigarette industry’s increasingly diversified marketing strategies^11^, further surveillance of industry e-cigarette marketing and other e-cigarette related content, including media channels used, source of the message, and message content, is needed. Research also is needed to better understand how consumers interpret different messages, experience different media channels, and respond in terms of their use behaviors and perceptions. In addition, considering the inadequate disclosure of harmful and potentially harmful chemicals in e-cigarettes^8^, transparent advertising content that accurately reflects these health risks is needed. Future research should examine the extent to which advertisements indicate the presence of harmful chemicals and assess their potential effects on consumer perceptions, intentions, and behaviors. Furthermore, regulatory and prevention efforts to reduce adult e-cigarette use may consider incorporating measures to reduce e-cigarette industry use of widely accessible media, including digital media, to circulate their advertising. Finally, tailored intervention efforts are needed to address key subpopulations likely to use e-cigarettes.

### Limitations

Study limitations include use of self-reported measures of advertisement and non-advertising content exposure, e-cigarette use outcomes, and tobacco use, which may be subject to recall bias (i.e. accuracy, reliability). For instance, participants may have underestimated their exposure to e-cigarette marketing content, particularly given that the exposure occurred on an infrequent basis. This bias may skew estimations towards null findings. Moreover, generalizability of our study findings may be limited given our focus on two countries and the sampling strategies used. Similar studies are warranted among other samples in the US, Israel and other countries, as well as among specific subgroups (e.g. those not reporting e-cigarette use). Further, the cross-sectional nature of this observational study does not allow causal inference. For example, it may be that people who intend to use e-cigarettes in the next year are more likely to notice e-cigarette ads. Finally, although we controlled for various relevant covariates, there are possible unknown confounders.

## CONCLUSIONS

Exposure to e-cigarette advertisements and non-advertising content was prevalent among US and Israeli adults. Study findings provide new insights regarding the types of exposures that may be most relevant for certain outcomes and that the context and content of this e-cigarette-related content is important in understanding the effects on e-cigarette use behaviors and related perceptions. Further surveillance is needed to monitor e-cigarette companies’ marketing activities and evaluate the potential impacts on e-cigarette related perceptions and use outcomes among adults across different regulatory contexts.

## Data Availability

The data presented in this study are available on request from the corresponding author. The data are not publicly available due to ethical reasons.
