# Supplementary material for: Exposure to e-cigarette advertisements and non-advertising content in relation to use behaviors and perceptions among US and Israeli adults
Source: Tob Prev Cessat. 2023 Nov 29;9:35. doi: 10.18332/tpc/173558 (PMC10685321; doi:10.18332/tpc/173558)
Supplement: Supplementary file 1 [file TPC-9-35-s1.pdf]

Supplemental Table 1a. Cross-sectional bivariate analyses examining exposure to e-cigarette promotions and covariates in relation to current e-cigarette use, use intentions, endorsement, and risk perceptions among US adults in 2021 (N=1,128)

	**Overall**	**Current e-cigarette use**			**E-cigarette use intentions**		**E-cigarette endorsement**		**Perceived addictiveness**		**Perceived harm**	
		**No**	**Yes**									
	**n (%) or M (SD)§**	**n (%) or M (SD)§**	**n (%) or M (SD)§**	**p**	**M (SD) or Coef.§**	**p**	**M (SD) or Coef.§**	**p**	**M (SD) or Coef.§**	**p**	**M (SD) or Coef.§**	**p**
**Overall**	1,128 (100)	929 (84.5)	170 (15.5)		1.76 (1.72)		2.65 (1.12)		5.42 (2.05)		5.71 (1.72)	
**Past-month e-cigarette ad exposure***												
Number of media channels (0-11)§	1.05 (1.69)	0.86 (1.55)	2.16 (2.06)	**<.001**	0.19	**<.001**	0.11	**<.001**	-0.01	.916	-0.06	.063
Any ad exposure	456 (42.5)	329 (36.4)	127 (75.2)	**<.001**	2.28 (2.06)	**<.001**	2.82 (1.18)	**<.001**	5.32 (2.00)	.160	5.55 (1.62)	**<.001**
No	617 (57.5)	575 (63.6)	42 (24.9)		1.39 (1.29)		2.52 (1.05)		5.51 (2.07)		5.87 (1.74)	
Digital media	233 (21.3)	151 (16.3)	82 (48.5)	**<.001**	2.56 (2.18)	**<.001**	2.90 (1.19)	**<.001**	4.99 (2.11)	**<.001**	5.40 (1.72)	**<.001**
No	861 (78.7)	774 (83.7)	87 (51.5)		1.54 (1.49)		2.58 (1.09)		5.53 (2.03)		5.80 (1.72)	
Traditional media	297 (28.0)	213 (23.6)	84 (52.2)	**<.001**	2.32 (2.13)	**<.001**	2.79 (1.16)	**.009**	5.63 (1.79)	.330	5.65 (1.51)	.280
No	765 (72.0)	688 (76.4)	77 (47.8)		1.53 (1.46)		2.59 (1.10)		5.32 (2.17)		5.75 (1.82)	
Retail settings	147 (13.4)	110 (11.9)	37 (21.9)	**<.001**	2.08 (1.85)	**.015**	2.97 (1.19)	**<.001**	5.03 (2.15)	**.015**	5.58 (1.70)	**<.001**
No	947 (86.6)	815 (88.1)	132 (78.1)		1.70 (1.69)		2.60 (1.10)		5.47 (2.04)		5.74 (1.73)	
**Past-month e-cigarette non-ad exposure**												
Number of media channels (0-4)§	0.38 (0.75)	0.31 (0.68)	0.80 (0.96)	**<.001**	0.213	**<.001**	0.13	**<.001**	-0.09	**.004**	-0.11	**<.001**
Any non-ad exposure	281 (26.3)	194 (21.5)	87 (52.4)	**<.001**	2.45 (2.11)	**<.001**	2.91 (1.22)	**<.001**	5.13 (2.05)	**.004**	5.41 (1.69)	**<.001**
No	786 (73.7)	707 (78.5)	79 (47.6)		1.50 (1.45)		2.55 (1.06)		5.54 (2.03)		5.86 (1.68)	
Movie, television, or theater	134 (12.3)	95 (10.3)	39 (23.2)	**<.001**	2.46 (2.17)	**<.001**	2.85 (1.27)	**.028**	5.25 (2.03)	.339	5.51 (1.72)	.144
No	955 (87.7)	826 (89.7)	129 (76.8)		1.66 (1.61)		2.62 (1.09)		5.43 (2.07)		5.74 (1.73)	
Radio, news podcasts	36 (3.3)	25 (2.7)	11 (6.6)	**.011**	2.12 (1.72)	.207	3.11 (1.18)	**.012**	3.94 (2.33)	**<.001**	4.69 (2.00)	**<.001**
No	1053 (96.7)	896 (97.3)	157 (93.5)		1.74 (1.71)		2.63 (1.11)		5.46 (2.03)		5.75 (1.71)	
Websites	103 (9.5)	63 (6.8)	40 (23.8)	**<.001**	2.50 (2.03)	**<.001**	3.01 (1.18)	**<.001**	4.80 (2.13)	**<.001**	5.19 (1.78)	**<.001**
No	986 (90.5)	858 (93.2)	128 (76.2)		1.68 (1.65)		2.61 (1.10)		5.47 (2.05)		5.77 (1.71)	
Social media	144 (13.2)	99 (10.8)	45 (26.8)	**<.001**	2.54 (2.21)	**<.001**	2.92 (1.18)	**<.001**	5.30 (1.89)	.513	5.42 (1.64)	**.029**
No	945 (86.8)	822 (89.3)	123 (73.2)		1.63 (1.58)		2.61 (1.10)		5.42 (2.09)		5.76 (1.74)	
**Past-month tobacco use status**												
E-cigarettes	170 (15.5)	0 (0)	170 (100)	**<.001**	4.54 (2.29)	**<.001**	2.92 (1.19)	**<.001**	5.28 (1.84)	.305	5.08 (1.59)	**<.001**
No	929 (84.5)	929 (100)	0 (0)		1.20 (0.79)		2.60 (1.10)		5.46 (2.08)		5.86 (1.71)	
Cigarettes	248 (22.9)	158 (17.2)	90 (53.9)	**<.001**	2.46 (2.05)	**<.001**	2.79 (1.15)	**.021**	5.20 (1.99)	.305	5.49 (1.67)	**.020**
No	836 (77.1)	759 (82.8)	77 (46.1)		1.53 (1.52)		2.60 (1.10)		5.48 (2.08)		5.79 (1.74)	
Other tobacco products ^	169 (15.7)	94 (10.3)	75 (44.6)	**<.001**	2.78 (2.21)	**<.001**	2.92 (1.18)	**<.001**	4.96 (2.07)	**<.001**	5.31 (1.68)	**<.001**
No	911 (84.4)	818 (89.7)	93 (55.4)		1.54 (1.50)		2.59 (1.10)		5.53 (2.03)		5.81 (1.71)	
**Demographics**												
Age – 18-45	156 (14.2)	115 (12.4)	41 (24.1)	**<.001**	2.29 (2.14)	**<.001**	2.51 (1.10)	.088	5.26 (2.12)	.593	5.69 (1.63)	.663
26-35	426 (38.8)	362 (39.0)	64 (37.7)		1.74 (1.65)		2.73 (1.14)		5.43 (1.99)		5.67 (1.72)	

36-45	517 (47.0)	452 (48.7)	65 (38.2)		1.61 (1.58)		2.63 (1.10)		5.45 (2.10)		5.77 (1.76)	
Gender – Female	545 (49.6)	468 (50.4)	77 (45.3)	.223	1.70 (1.69)	.246	2.63 (1.07)	.528	5.55 (2.07)	**.027**	5.91 (1.65)	**<.001**
Male	554 (50.4)	461 (49.6)	93 (54.7)		1.82 (1.75)		2.67 (1.16)		5.27 (2.04)		5.52 (1.78)	
Sexual orientation – Heterosexual	959 (87.3)	822 (88.6)	137 (80.6)	**.004**	1.70 (1.66)	**.004**	2.65 (1.10)	.826	5.39 (2.05)	.294	5.74 (1.70)	.269
Sexual orientation minority	139 (12.7)	106 (11.4)	33 (19.4)		2.16 (2.02)		2.63 (1.22)		5.59 (2.09)		5.57 (1.88)	
Education level <College degree	623 (56.7)	512 (55.1)	111 (65.3)	**.014**	1.91 (1.87)	**<.001**	2.75 (1.06)	**<.001**	5.19 (2.22)	**<.001**	5.59 (1.84)	**<.001**
≥College degree	476 (43.3)	417 (44.9)	59 (34.7)		1.56 (1.47)		2.52 (1.17)		5.72 (1.77)		5.89 (1.53)	
Relationship status – Married/cohabiting	590 (53.7)	507 (54.6)	83 (48.8)	.167	1.67 (1.64)	.063	2.63 (1.12)	.596	5.61 (1.96)	**<.001**	5.83 (1.66)	**.029**
Other	509 (46.3)	422 (45.4)	87 (51.2)		1.86 (1.79)		2.67 (1.11)		5.19 (2.15)		5.59 (1.79)	
Children under 18 in the home	513 (46.7)	437 (47.0)	76 (44.7)	.575	1.72 (1.70)	.492	2.69 (1.10)	.317	5.39 (2.10)	.676	5.74 (1.78)	.756
No children under 18 in the home	586 (53.3)	492 (53.0)	94 (55.3)		1.79 (1.73)		2.62 (1.13)		5.44 (2.01)		5.70 (1.67)	

Notes: § For continuous variables, M (SD) reported for first 3 columns and Coeff for other columns. * Digital Media: websites, social media, direct mail, email. Retail: inside or outside tobacco shop, vape shop. Traditional Media: television, radio, newspapers/magazines, posters/billboards. ^ Other tobacco includes heated tobacco products, hookah, cigar, pipe, and smokeless tobacco. Boldface indicates p<.05.

Supplemental Table 1a. Cross-sectional bivariate analyses examining exposure to e-cigarette promotions and covariates in relation to current e-cigarette use, use intentions, endorsement, and risk perceptions among Israeli adults in 2021 (N=1,094)

	Overall	Current e-cigarette use No	Current e-cigarette use Yes		E-cigarette use intentions		E-cigarette endorsement		Perceived addictiveness		Perceived harm	
	n (%) or M (SD)§	n (%) or M (SD)§	n (%) or M (SD)§	p	M (SD) or Coef.§	p	M (SD) or Coef.§	p	M (SD) or Coef.§	p	M (SD) or Coef.§	p
**Overall**	**1094 (100)**	816 (74.8)	275 (25.2)		2.16 (1.87)		2.99 (1.10)		5.10 (1.99)		5.58 (1.75)	
**Past-month e-cigarette ad exposure***												
Number of media channels (0-11)§	1.17 (1.66)	0.87 (1.43)	2.06 (1.97)	**<.001**	0.32	**<.001**	0.07	**.025**	-0.07	**.027**	-0.11	**<.001**
Any ad exposure	529 (49.8)	311 (39.3)	218 (80.4)	**<.001**	2.88 (2.10)	**<.001**	3.10 (1.11)	**<.001**	4.77 (2.04)	**<.001**	5.20 (1.86)	**<.001**
No	533 (50.2)	480 (60.7)	53 (19.6)		1.47 (1.27)		2.89 (1.06)		5.52 (1.80)		6.00 (1.48)	
Digital media	394 (36.1)	225 (27.6)	169 (61.5)	**<.001**	2.94 (2.10)	**<.001**	3.15 (1.07)	**<.001**	4.84 (1.99)	**<.001**	5.26 (1.83)	**<.001**
No	697 (63.9)	591 (72.4)	106 (38.6)		1.73 (1.57)		2.90 (1.10)		5.24 (1.98)		5.75 (1.68)	
Traditional media	233 (22.3)	127 (15.9)	106 (42.7)	**<.001**	3.09 (2.17)	**<.001**	3.13 (1.15)	**.033**	4.85 (1.99)	**.025**	5.26 (1.77)	**<.001**
No	812 (77.7)	670 (84.1)	142 (57.3)		1.81 (1.61)		2.95 (1.08)		5.18 (1.99)		5.71 (1.72)	
Retail settings	219 (20.1)	131 (16.1)	88 (32.0)	**<.001**	2.89 (2.05)	**<.001**	2.99 (1.11)	.923	4.73 (2.00)	**<.001**	5.09 (1.92)	**<.001**
No	872 (79.9)	685 (84.0)	187 (68.0)		1.98 (1.78)		2.99 (1.09)		5.19 (1.98)		5.70 (1.69)	
**Past-month e-cigarette non-ad exposure**												
Number of media channels (0-4)§	0.64 (0.87)	0.45 (0.75)	1.19 (0.95)	**<.001**	0.386	**<.001**	0.05	.115	-0.08	**.006**	-0.15	**<.001**
Any non-ad exposure	463 (43.4)	258 (32.5)	205 (75.1)	**<.001**	3.02 (2.14)	**<.001**	3.08 (1.13)	**.015**	4.84 (2.02)	**<.001**	5.17 (1.86)	**<.001**
No	603 (56.6)	535 (67.5)	68 (24.9)		1.53 (1.35)		2.92 (1.06)		5.37 (1.89)		5.92 (1.54)	
Movie, television, or theater	133 (12.2)	77 (9.4)	56 (20.4)	**<.001**	2.93 (1.99)	**<.001**	3.06 (1.20)	.443	4.56 (2.12)	**<.001**	5.05 (2.05)	**<.001**
No	958 (87.8)	739 (90.6)	219 (79.6)		2.06 (1.83)		2.98 (1.08)		5.17 (1.96)		5.65 (1.70)	
Radio, news podcasts	90 (8.3)	50 (6.1)	40 (14.6)	**<.001**	3.27 (2.08)	**<.001**	2.86 (1.10)	.250	4.74 (1.92)	.079	4.84 (1.83)	**<.001**
No	1001 (91.8)	766 (93.9)	235 (85.5)		2.06 (1.82)		3.00 (1.10)		5.13 (1.99)		5.64 (1.73)	
Websites	194 (17.8)	101 (12.4)	93 (33.8)	**<.001**	3.15 (2.18)	**<.001**	3.02 (1.12)	.693	4.87 (2.00)	.080	5.13 (1.92)	**<.001**
No	897 (82.2)	715 (87.6)	182 (66.2)		1.95 (1.73)		2.99 (1.09)		5.15 (1.99)		5.67 (1.70)	
Social media	278 (25.5)	140 (17.2)	138 (50.2)	**<.001**	3.22 (2.22)	**<.001**	3.16 (1.12)	**.003**	5.06 (1.97)	.749	5.47 (1.65)	.250
No	813 (74.5)	676 (82.8)	137 (49.8)		1.81 (1.59)		2.93 (1.08)		5.11 (2.00)		5.61 (1.79)	
**Past-month tobacco use status**												
E-cigarettes	275 (25.2)	0 (0)	275 (100)	**<.001**	4.08 (2.12)	**<.001**	3.29 (1.01)	**<.001**	4.76 (1.94)	**<.001**	4.98 (1.71)	**<.001**
No	816 (74.8)	816 (100)	0 (0)		1.52 (1.24)		2.89 (1.11)		5.22 (1.99)		5.78 (1.72)	
Cigarettes	428 (39.3)	218 (26.7)	210 (76.6)	**<.001**	3.27 (2.13)	**<.001**	3.13 (1.02)	**<.001**	4.95 (1.84)	**.048**	5.34 (1.66)	**<.001**
No	662 (60.7)	598 (73.3)	64 (23.4)		1.45 (1.24)		2.90 (1.14)		5.20 (2.08)		5.73 (1.79)	
Other tobacco products ^	354 (32.5)	166 (20.3)	188 (68.6)	**<.001**	3.15 (2.13)	**<.001**	3.14 (1.07)	**<.001**	4.61 (2.01)	**<.001**	4.94 (1.93)	**<.001**
No	736 (67.5)	650 (79.7)	86 (31.4)		1.69 (1.53)		2.92 (1.10)		5.34 (1.93)		5.89 (1.57)	
**Demographics**												
Age – 18-45	383 (35.1)	284 (34.8)	99 (36)	**<.001**	2.06 (1.83)	.360	2.97 (1.10)	.569	4.81 (2.17)	**<.001**	5.42 (1.91)	.097
26-35	392 (35.9)	293 (35.9)	99 (36)		2.19 (1.86)		2.97 (1.07)		5.36 (1.88)		5.65 (1.69)	

36-45	316 (29.0)	239 (29.3)	77 (28)		2.26 (1.94)		3.05 (1.12)		5.12 (1.84)		5.67 (1.62)	
Gender – Female	555 (50.9)	433 (53.1)	122 (44.4)	**.013**	1.97 (1.78)	**<.001**	2.96 (1.06)	.266	5.24 (1.99)	**<.001**	5.84 (1.63)	**<.001**
Male	536 (49.1)	383 (46.9)	153 (55.6)		2.36 (1.95)		3.03 (1.13)		4.96 (1.98)		5.30 (1.83)	
Sexual orientation – Heterosexual	899 (82.4)	676 (82.8)	223 (81.1)	.509	2.16 (1.85)	.786	2.97 (1.09)	.100	5.18 (1.93)	**<.001**	5.66 (1.69)	**<.001**
Sexual orientation minority	192 (17.6)	140 (17.2)	52 (18.9)		2.20 (1.98)		3.11 (1.10)		4.70 (2.22)		5.16 (1.98)	
Education level <College degree	469 (43.0)	350 (42.9)	119 (43.3)	.912	2.20 (1.93)	.600	2.97 (1.12)	.514	4.81 (2.11)	**<.001**	5.49 (1.82)	.147
≥College degree	622 (57.0)	466 (57.1)	156 (56.7)		2.14 (1.83)		3.01 (1.08)		5.31 (1.87)		5.64 (1.70)	
Relationship status – Married/cohabiting	583 (53.4)	417 (51.1)	166 (60.4)	**.008**	2.36 (1.97)	**<.001**	2.99 (1.10)	.968	5.20 (1.91)	.084	5.60 (1.70)	.626
Other	508 (46.6)	399 (48.9)	109 (39.6)		1.94 (1.72)		2.99 (1.09)		4.98 (2.08)		5.55 (1.82)	
Children under 18 in the home	595 (54.5)	432 (52.9)	163 (59.3)	.068	2.29 (2.01)	**.012**	3.00 (1.08)	.722	5.19 (1.93)	.087	5.65 (1.68)	.105
No children under 18 in the home	496 (45.5)	384 (47.1)	112 (40.7)		2.01 (1.68)		2.98 (1.12)		4.98 (2.05)		5.48 (1.83)	

Notes: § For continuous variables, M (SD) reported for first 3 columns and Coeff for other columns. * Digital Media: websites, social media, direct mail, email. Retail: inside or outside tobacco shop, vape shop. Traditional Media: television, radio, newspapers/magazines, posters/billboards. ^ Other tobacco includes heated tobacco products, hookah, cigar, pipe, and smokeless tobacco. Boldface indicates p<.05.

Supplemental Table 2a. Cross-sectional multivariable regression analyses examining exposure to e-cigarette promotions and covariates in relation to current e-cigarette use, use intentions, endorsement, and risk perceptions among US adults in 2021 (N=1,128)

	**Current e-cigarette use**		**E-cigarette use intentions**		**E-cigarette endorsement**		**Perceived addictiveness**		**Perceived harm**	
	**aOR**	**95% CI**	**β**	**95% CI**	**β**	**95% CI**	**β**	**95% CI**	**β**	**95% CI**
**Past-month e-cigarette ad exposure**										
Digital media (Ref: No)	**2.75**	**1.66, 4.55**	0.18	-0.06, 0.41	-0.01	-0.23, 0.20	-0.32	-0.72, 0.08	-0.03	-0.37, 0.31
Traditional media (Ref: No)	**2.54**	**1.66, 3.89**	0.12	-0.06, 0.30	0.01	-0.16, 0.18	**0.68**	**0.37, 0.99**	0.09	-0.17, 0.36
Retail settings (Ref: No)	0.58	0.32, 1.07	-0.13	-0.38, 0.12	0.19	-0.04, 0.43	**-0.46**	**-0.89, -0.03**	0.13	-0.24, 0.49
**Past-month e-cigarette non-ad exposure**										
Movie, television, or theater (Ref: No)	1.32	0.76, 2.30	**0.25**	**0.00, 0.49**	-0.01	-0.23, 0.21	0.13	-0.29, 0.55	0.13	-0.22, 0.49
Radio, news podcasts (Ref: No)	1.16	0.46, 2.92	**-0.45**	**-0.88, -0.02**	0.14	-0.27, 0.54	**-1.21**	**-1.95, -0.48**	**-0.97**	**-1.59, -0.35**
Websites (Ref: No)	1.44	0.78, 2.66	-0.14	-0.43, 0.15	0.18	-0.09, 0.45	-0.49	-1.00, 0.02	-0.41	-0.84, 0.02
Social media (Ref: No)	0.77	0.43, 1.38	**0.27**	**0.01, 0.53**	0.17	-0.07, 0.41	**0.46**	**0.01, 0.91**	0.01	-0.38, 0.38
**Current tobacco use status**										
E-cigarettes (Ref: No)	--	--	**3.22**	**3.00, 3.45**	0.18	-0.03, 0.39	-0.07	-0.46, 0.33	**-0.73**	**-1.06, -0.39**
Cigarettes (Ref: No)	**3.17**	**2.04, 4.94**	-0.04	-0.24, 0.16	0.02	-0.16, 0.21	-0.06	-0.40, 0.28	0.04	-0.25, 0.33
Other tobacco products* (Ref: No)	**3.77**	**2.35, 6.03**	0.07	-0.17, 0.30	0.18	-0.04, 0.40	**-0.42**	**-0.82, -0.01**	-0.12	-0.46, 0.22
**Demographics**										
Age (Ref: 36-45)										
18-25	**3.62**	**2.05, 6.40**	0.12	-0.11, 0.35	**-0.22**	**-0.43, -0.01**	-0.02	-0.41, 0.37	0.18	-0.16, 0.51
26-35	**1.61**	**1.02, 2.53**	0.03	-0.13, 0.19	0.09	-0.06, 0.24	0.06	-0.22, 0.34	0.02	-0.22, 0.25
Female (Ref: Male)	0.96	0.64, 1.44	-0.04	-0.19, 0.10	-0.02	-0.16, 0.12	**0.26**	**0.00, 0.51**	**0.38**	**0.16, 0.59**
Sexual orientation minority (Ref: Heterosexual)	**1.82**	**1.07, 3.09**	0.06	-0.16, 0.29	-0.11	-0.32, 0.10	0.23	-0.16, 0.62	-0.08	-0.40, 0.25
Education <College (Ref: ≥College)	0.99	0.65, 1.51	0.12	-0.03, 0.27	**0.29**	**0.15, 0.43**	**-0.56**	**-0.83, -0.30**	**-0.35**	**-0.57, -0.13**

Notes: * Other tobacco includes heated tobacco products, hookah, cigar, pipe, and smokeless tobacco. Boldface indicates p<.05.

Supplemental Table 2b. Cross-sectional multivariable regression analyses examining exposure to e-cigarette promotions and covariates in relation to current e-cigarette use, use intentions, endorsement, and risk perceptions among Israeli adults in 2021 (N=1,094)

	**Current e-cigarette use**		**E-cigarette use intentions**		**E-cigarette endorsement**		**Perceived addictiveness**		**Perceived harm**	
	**aOR**	**95% CI**	**β**	**95% CI**	**β**	**95% CI**	**β**	**95% CI**	**β**	**95% CI**
**Past-month e-cigarette ad exposure**										
Digital media (Ref: No)	**1.58**	**1.04, 2.39**	0.12	-0.11, 0.34	**0.24**	**0.07, 0.42**	-0.28	-0.59, 0.03	-0.21	-0.47, 0.06
Traditional media (Ref: No)	**1.73**	**1.14, 2.62**	**0.32**	**0.08, 0.56**	0.08	-0.11, 0.26	-0.03	-0.36, 0.30	-0.02	-0.31, 0.26
Retail settings (Ref: No)	0.67	0.42, 1.06	-0.05	-0.31, 0.20	-0.07	-0.27, 0.12	-0.14	-0.49, 0.21	-0.13	-0.43, 0.17
**Past-month e-cigarette non-ad exposure**										
Movie, television, or theater (Ref: No)	0.83	0.49, 1.42	0.06	-0.24, 0.35	-0.10	-0.33, 0.13	-0.31	-0.71, 0.09	-0.27	-0.61, 0.07
Radio, news podcasts (Ref: No)	0.87	0.48, 1.57	0.27	-0.08, 0.61	**-0.28**	**-0.55, -0.01**	-0.08	-0.56, 0.40	-0.28	-0.69, 0.13
Websites (Ref: No)	1.10	0.70, 1.74	0.13	-0.13, 0.39	-0.15	-0.35, 0.05	0.08	-0.28, 0.45	-0.04	-0.35, 0.27
Social media (Ref: No)	**2.46**	1.64, 3.70	**0.34**	**0.11, 0.58**	-0.01	-0.19, 0.17	**0.41**	**0.09, 0.73**	**0.34**	**0.06, 0.61**
**Current tobacco use status**										
E-cigarettes (Ref: No)	--	--	**1.71**	**1.47, 1.96**	**0.34**	**0.15, 0.53**	-0.22	-0.56, 0.12	**-0.46**	**-0.75, -0.17**
Cigarettes (Ref: No)	**5.00**	**3.41, 7.31**	**0.81**	**0.60, 1.02**	0.05	-0.12, 0.21	0.05	-0.24, 0.34	0.10	-0.15, 0.34
Other tobacco products* (Ref: No)	**5.29**	**3.58, 7.79**	0.14	-0.09, 0.36	0.07	-0.11, 0.24	**-0.56**	**-0.87, -0.25**	**-0.54**	**-0.80, -0.27**
**Demographics**										
Age (Ref: 36-45)										
18-25	0.84	0.53, 1.34	**-0.26**	**-0.49, -0.04**	-0.08	-0.25, 0.10	-0.14	-0.46, 0.17	-0.1	-0.36, 0.17
26-35	0.93	0.59, 1.45	-0.10	-0.32, 0.11	-0.08	-0.25, 0.09	0.23	-0.07, 0.52	-0.06	-0.31, 0.20
Female (Ref: Male)	1.22	0.84, 1.77	-0.09	-0.26, 0.09	-0.06	-0.20, 0.07	0.07	-0.17, 0.32	**0.38**	**0.17, 0.59**
Sexual orientation minority (Ref: Heterosexual)	1.16	0.72, 1.87	0.07	-0.16, 0.30	**0.20**	**0.02, 0.38**	-0.26	-0.58, 0.06	**-0.35**	**-0.62, -0.08**
Education <College (Ref: ≥College)	1.08	0.74, 1.58	0.09	-0.09, 0.28	-0.06	-0.20, 0.08	**-0.43**	**-0.68, -0.17**	-0.12	-0.33, 0.10

Notes: * Other tobacco includes heated tobacco products, hookah, cigar, pipe, and smokeless tobacco. Boldface indicates p<.05.

Supplemental Table 3a. Sensitivity Analyses: Cross-sectional multivariable regression analyses examining exposure to e-cigarette promotions per number of channels and covariates in relation to current e-cigarette use, use intentions, endorsement, and risk perceptions among US adults in 2021 (N=1,128)

	**Current e-cigarette use**		**E-cigarette use intentions**		**E-cigarette endorsement**		**Perceived addictiveness**		**Perceived harm**	
	**aOR**	**95% CI**	**β**	**95% CI**	**β**	**95% CI**	**β**	**95% CI**	**β**	**95% CI**
**Ad exposure - # media channels (0-10)**	**1.29**	**1.15, 1.44**	-0.04	-0.09, 0.01	0.02	-0.03, 0.06	**0.1**	**0.01, 0.19**	0.04	-0.03, 0.11
**Non-ad exposure - # media channels (0-4)**	1.09	0.84, 1.41	**0.17**	**0.06, 0.29**	**0.12**	**0.01, 0.23**	**-0.28**	**-0.48, -0.08**	**-0.17**	**-0.34, -0.01**
**Current tobacco use status**										
E-cigarettes (Ref: No)	--	--	**3.23**	**3.00, 3.45**	0.14	-0.06, 0.35	-0.03	-0.42, 0.35	**-0.72**	**-1.04, -0.40**
Cigarettes (Ref: No)	**3.28**	**2.15, 5.02**	-0.01	-0.20, 0.18	0.02	-0.16, 0.20	-0.05	-0.39, 0.28	0.01	-0.28, 0.28
Other tobacco products* (Ref: No)	**3.86**	**2.46, 6.04**	0.06	-0.17, 0.29	0.19	-0.03, 0.40	**-0.42**	**-0.81, -0.02**	-0.11	-0.43, 0.22
**Demographics**										
Age (Ref: 36-45)										
18-25	**3.35**	**1.95, 5.76**	0.11	-0.11, 0.34	**-0.22**	**-0.43, -0.01**	0.04	-0.35, 0.43	0.18	-0.15, 0.51
26-35	1.42	0.92, 2.20	0.03	-0.13, 0.19	0.09	-0.06, 0.24	0.06	-0.21, 0.34	0.01	-0.22, 0.24
Female (Ref: Male)	0.96	0.66, 1.42	-0.07	-0.22, 0.07	-0.02	-0.16, 0.11	0.23	-0.02, 0.49	**0.37**	**0.16, 0.58**
Sexual orientation minority (Ref: Heterosexual)	**1.80**	**1.08, 3.01**	0.09	-0.13, 0.32	-0.12	-0.32, 0.09	0.22	-0.16, 0.61	-0.05	-0.37, 0.27
Education <College (Ref: ≥College)	0.94	0.63, 1.41	0.14	-0.01, 0.29	**0.26**	**0.12, 0.40**	**-0.58**	**-0.84, -0.32**	**-0.34**	**-0.55, -0.12**

Notes: * Other tobacco includes heated tobacco products, hookah, cigar, pipe, and smokeless tobacco. Boldface indicates p<.05.

Supplemental Table 3b. Sensitivity Analyses: Cross-sectional multivariable regression analyses examining exposure to e-cigarette promotions per number of channels and covariates in relation to current e-cigarette use, use intentions, endorsement, and risk perceptions among Israeli adults in 2021 (N=1,094)

	**Current e-cigarette use**		**E-cigarette use intentions**		**E-cigarette endorsement**		**Perceived addictiveness**		**Perceived harm**	
	**aOR**	**95% CI**	**β**	**95% CI**	**β**	**95% CI**	**β**	**95% CI**	**β**	**95% CI**
**Ad exposure - # media channels (0-10)**	1.12	0.99, 1.26	0.06	-0.01, 0.12	0.03	-0.02, 0.08	0.01	-0.08, 0.10	0.02	-0.06, 0.10
**Non-ad exposure - # media channels (0-4)**	**1.44**	**1.14, 1.82**	**0.25**	**0.11, 0.38**	-0.06	-0.17, 0.04	-0.03	-0.21, 0.15	-0.09	-0.25, 0.06
**Current tobacco use status**										
E-cigarettes (Ref: No)	--	--	**1.84**	**1.60, 2.07**	**0.35**	**0.17, 0.53**	-0.14	-0.46, 0.17	**-0.43**	**-0.71, -0.16**
Cigarettes (Ref: No)	**4.89**	**3.43, 6.98**	**0.83**	**0.62, 1.03**	0.10	-0.06, 0.25	0.04	-0.24, 0.32	0.07	-0.17, 0.31
Other tobacco products* (Ref: No)	**4.41**	**3.08, 6.31**	0.12	-0.10, 0.34	0.03	-0.14, 0.20	**-0.64**	**-0.94, -0.34**	**-0.63**	**-0.89, -0.37**
**Demographics**										
Age (Ref: 36-45)										
18-25	0.93	0.59, 1.44	**-0.27**	**-0.49, -0.04**	-0.09	-0.26, 0.09	-0.09	-0.40, 0.22	-0.13	-0.39, 0.14
26-35	0.95	0.63, 1.45	-0.08	-0.30, 0.13	-0.08	-0.25, 0.08	0.23	-0.06, 0.52	-0.07	-0.32, 0.19
Female (Ref: Male)	1.26	0.89, 1.79	-0.07	-0.24, 0.11	-0.03	-0.17, 0.10	0.10	-0.14, 0.34	**0.39**	**0.18, 0.60**
Sexual orientation minority (Ref: Heterosexual)	1.08	0.69, 1.69	0.08	-0.15, 0.31	0.16	-0.02, 0.33	-0.28	-0.59, 0.03	**-0.36**	**-0.63, -0.09**
Education <College (Ref: ≥College)	1.11	0.78, 1.58	0.10	-0.08, 0.28	-0.06	-0.20, 0.08	**-0.43**	**-0.67, -0.18**	-0.10	-0.31, 0.12

Notes: * Other tobacco includes heated tobacco products, hookah, cigar, pipe, and smokeless tobacco. Boldface indicates p<.05.

© 2023 Duan Z. et al
